# Leukotriene B_4_ loaded in microspheres regulate the expression of genes related to odontoblastic differentiation and biomineralization by dental pulp stem cells

**DOI:** 10.1186/s12903-022-02083-8

**Published:** 2022-02-23

**Authors:** Francine Lorencetti da Silva, Giuliana de Campos Chaves Lamarque, Fernanda Maria Machado Pereira Cabral de Oliveira, Paulo Nelson-Filho, Léa Assed Bezerra da Silva, Raquel Assed Bezerra Segato, Lúcia Helena Faccioli, Francisco Wanderley Garcia Paula-Silva

**Affiliations:** 1 Universidade de Rio Verde, Rio Verde, GO Brazil; 2 Department of Pediatric Clinics, Faculty of Dentistry of Ribeirão Preto, University of São Paulo, Ribeirão Prêto, SP Brazil; 3 Departamento de Análises Clínicas, Toxicológicas e Bromatológicas da Faculdade de Ciências Farmacêuticas de Ribeirão Preto, Universidade de São Paulo, Ribeirão Prêto, SP Brazil

**Keywords:** Dental pulp stem cells, Leukotriene, Microspheres, Odontoblast, Differentiation

## Abstract

**Background:**

Leukotriene B_4_ (LTB_4_) is a potent lipid mediator that stimulate the immune response. Because dental pulp inflammation and dentin repair are intrinsically related responses, the aim of this research was to investigate the potential of LTB_4_ in inducing differentiation of dental pulp stem cells.

**Methods:**

Microspheres (MS) loaded with LTB_4_ were prepared using an oil emulsion solvent extraction evaporation process and sterility, characterization, efficiency of LTB_4_ encapsulation and in vitro LTB_4_ release assay were investigated. Mouse dental pulp stem cells (OD-21) were stimulated with soluble LTB_4_ or MS loaded with LTB_4_ (0.01 and 0.1 μM). Cytotoxicity and cell viability was determined by lactate dehydrogenase and methylthiazol tetrazolium assays. Gene expression were measured by quantitative reverse transcription polymerase chain reaction after 3, 6, 24, 48 and 72 h. Mineralized nodule formation was assessed after 28 days of OD-21 cell stimulation with LTB_4_ in mineralized media or not. Groups were compared using one-way ANOVA test followed by Dunnett’s post-test (α = 0.05).

**Results:**

Treatment with LTB_4_ or MS loaded with LTB_4_ (0.01 and 0.1 µm-μM) were not cytotoxic to OD-21 cells. Treatment with LTB_4_ modulated the expression of the *Ibsp* (integrin binding sialoprotein) and *Runx2* (runt-related transcription factor 2) genes differently depending on the experimental period analyzed. Interestingly LTB_4_ loaded in microspheres (0.1 μM) allowed long term dental pulp cell differentiation and biomineralization.

**Conclusion:**

LTB_4_, soluble or loaded in MS, were not cytotoxic and modulated the expression of the *Ibsp* and *Runx2* genes in cultured OD-21 cells. When LTB_4_ was incorporated into MS, odontoblast differentiation and mineralization was induced in long term culture.

## Introduction

Pulp and dentin are closely related tissues, being assembled as a single unit, the dentin-pulp complex, which is a strategic and dynamic barrier in face of injuries suffered by teeth, being caries the most common cause of injury to this complex [1, 2]. Odontoblasts, located around the pulp, are the first to have contact with pathogens, producing dentine matrix in order to protect the pulp [3, 4]. However, deep cavity preparations or dental pulp exposure can disrupt the integrity of the dentin-pulp complex and may cause odontoblast cell death [5]. Thus, the regeneration of these tissues occurs through stimulation and proliferation of mesenchymal progenitor cells, which are attracted to the injury site to differentiate into odontoblast-like cells and produce reparative dentin [6, 7].

Response to infection that occurs in the dental pulp is a complex molecular reaction that aims to eliminate the foreign pathogen. Cells and tissues at the injury site express receptors that recognize pathogenic signals, such as lipopolysaccharides, lipoteichoic acids and bacterial DNA [8]. In response to that, several inflammatory mediators are produced locally to orchestrate the immune response. Among those are the eicosanoids, a class of lipid mediators that are synthesized from arachidonic acid through the action of cyclooxygenases or lipoxygenases to produce prostaglandins and thromboxanes or leukotrienes (LT) and lipoxins, respectively [9, 10]. In the presence of FLAP (5-lipoxygenase activating protein), a nuclear protein associated with the membrane, the enzyme 5-LO is activated and oxidizes arachidonic acid, converting it to 5S-hydroxyperoeicosatetraenoic acid (5S-HpETE), which is further reduced by the enzyme peroxidase to 5S acid-hydroxyieicositetraenoic (5S-HETE) or is converted into LTA4, which, by the action of LTA4 hydrolase, results in LTB_4_ production [11].

Leukotriene B_4_ (LTB_4_) is a potent inflammatory mediator that also stimulates the immune response, induces the recruitment of phagocytes and potentiates the ingestion and death of pathogens, being one of the most recognized neutrophil activators, modulating the release of cytokines and increasing vascular permeability [12–14]. LTB_4_ binds either to high affinity receptor (BLT1), mainly in leukocytes, or to low affinity receptor (BLT2) [15]. However, soluble LTB_4_ present a short half-life and is rapidly degraded [16]. As a therapeutical strategy, the use of microspheres could preserve the biological activity and stability of the mediator for prolonged periods [13, 17, 18]. However, studies are lacking to investigate the role of these lipid mediators in dental pulp cell behavior, especially through the synthesis and deposition of dentinal matrix in undifferentiated cells. Therefore, the objective of this study was to investigate if LTB_4_ loaded in microspheres would induce odontoblastic cell differentiation and biomineralization. The null hypothesis of this study was that LTB_4_ did not impact odontoblast cell differentiation and function.

## Material and methods

### Preparation of microspheres

Microspheres (MS) were prepared as a pharmacological strategy using an oil-in-water emulsion solvent extraction-evaporation process [13, 19]. Briefly, LTB_4_ (CAYM-14010; Cayman Chemical Company, Michigan, USA) was dissolved in absolute ethanol (100 µg/mL). Then, 0.3 mL of the organic phase, equivalent to 3 × 10^−5^ M of the LTB_4_ solution was added to 10 mL of methylene chloride supplemented with 30 mg of 50:50 poly (lactic-co-glycolic acid) (PLGA) (Boehringer Ingelheim, Germany). Next, 40 mL of 3% polyvinyl alcohol (3% w/v PVA) (Sigma-Aldrich CO., St. Louis, MO, USA) were added and the mixture was mechanically stirred at 600 rpm for 4 h (RW-20; Ika®-Werke GmbH & CO. KG, Staufen, Germany). Microspheres were washed (3x) with deionized water (Milli-Q®, Merck Millipore, Darmstadt, Germany), lyophilized, and stored at − 20 °C until use.

### LPS contamination tests

For sterility test small microsphere aliquots were diluted in 500 µL of 1 × PBS (phosphate buffered saline) and 100 µL of solution was spread on Brain Heart Infusion (BHI)-Agar medium and kept in an incubator at 37 °C for 24 h to detect microbial contamination.

Microspheres were tested for LPS contamination using the Limulus Amebocyte Lysate (LAL) QCL-1000™ kit (Lonza Walkersville, Inc., Olten, Switzerland) according to the manufacturer’s instructions. To obtain the standard curve, the serial dilution regime was performed, starting from 1.0 EU/mL of *E. coli* endotoxin 0111: B4 (E50-640). Optical density was analyzed using a μQuantTM spectrophotometer at a wavelength of 405 ηm (BioTek® Instruments Inc., Winooski, USA), with KC4™ Data Analysis Software (BioTek® Instruments Inc.), in order to determine the concentration of endotoxin units/ml of solution containing microspheres (EU/ml).

### Characterization of microspheres

Size distribution of MS was determined using a LS 13 320 Laser Diffraction Particle Size Analyzer (Beckman Coulter, USA). Samples (1 mg) of either unloaded-MS or LTB_4_ -loaded MS was dispersed in 0.4 mL of purified sterile water and then analyzed at 25 °C. Zeta potential of MS was determined using a Zetasizer Nano (Malvern Instruments, England). Each sample was prepared dispersing 1 mg of unloaded-MS or LTB_4_-loaded MS in 0.4 mL of purified water containing 10 mM NaCl and then analyzed at 25 °C. Morphology of MS samples was assessed by scanning electron microscopy (SEM) using a FEI Inspect S 50 scanning microscope (FEI; Oregon, USA).

### Efficiency of LTB_4_ encapsulation in MS

For calculation of encapsulation efficiency, samples of LTB_4_-loaded MS (4 mg) were dissolved in 1 mL of acetonitrile/ethanol (7:3 v/v), to disrupt the MS structure. The solvent was then evaporated off in a vacuum concentrator centrifuge for 4 h, and the residue was reconstituted in 100 μL of methanol. Then, the supernatants were transferred to appropriate vials for determination of the concentration of LTB_4_ by a competition enzyme immunoassay, according to manufacturer's instructions (EIA, Amershan Biosciences, Piscataway, NJ, USA). Quantification in μM was accomplished using calibration curve containing LTB_4_ synthetic standards (Cayman Chemical, Ann Arbor, MI, USA).

### In vitro LTB_4_ release assay

The release kinetics of LTB_4_ from LTB_4_-MS were monitored in vitro. LTB_4_ (4 mg) was suspended in 1 mL of PBS/ethanol (50:50, v/v), pH 7.4, and incubated at 37 °C on a rotating incubator. At each time point 6, 12, 18, 24, 30, 36, 42, 48 and 54 h of rotation, the suspension was centrifuged and the supernatant was collected for assay of LTB_4_ concentration, then 1 mL of fresh PBS/ethanol was added to the flask containing the LTB_4_-MS and the experiment was continued.

The supernatants were transferred to appropriate vials for determination of the concentration of LTB_4_ by a competition enzyme immunoassay, according to manufacturer's instructions (EIA, Amersham Biosciences, Piscataway, NJ, USA). Quantification was accomplished using calibration curve containing LTB_4_ synthetic standards (Cayman Chemical, Ann Arbor, MI, USA).

### OD-21 cell culture

Murine immortalized undifferentiaded dental pulp cells (OD-21) were maintained in DMEM supplemented with 10% fetal bovine serum (FBS) (Gibco, Grand Island, NY, USA) and 1% Penicilin/Streptomicin (Gibco) in an incubator at 37 °C and 5% CO_2_ For the experiments, 1 × 10^5^ cells/well were plated into 48-well cell culture plates (Cell Wells, Corning Glass Workers, NY, USA) using DMEM without FBS and cells were left overnight for attachment.

Next, the culture medium was removed; wells were washed with phosphate buffered saline (PBS) and 300  µL LTB_4_-loaded MS or soluble LTB_4_ were added to each well. The experiments were done in duplicate and the stimuli were maintained for 3, 6, 24, 48 and 72 h for short term experiments or 28 days for long term biomineralization assay.

### Cytotoxicity: lactate dehydrogenase (LDH) assay

For cytotoxicity assessment, cells were plated in serum-free medium, at a concentration of 1 × 10^5^ cells per well, kept in an incubator at 37 °C and 5% CO_2_ for 12 h (*overnight*). After this period, cultures were stimulated with different concentrations of soluble LTB_4_ or microspheres with or without LTB_4_ at 0.01 μM e 0.1 μM, for 24 h. Next, 50 µL of the supernatant was collected and transferred to a new 96-well plate with a transparent, flat bottom and 50 µL of the CytoTox 96® Reagent was added to each sample. The plate was then covered with foil to protect against light and the samples incubated at 25 °C for 30 min. After this period, 50 μL of the Stop Solution was added to each well. The absorbance was measured at 490 nm with a spectrophotometer (mQuanti, Bio-Tek Instruments, Inc., Winooski, VT, USA). As positive control, 10 × Lysis Solution was added to the cells, 45 min prior to adding CytoTox 96® Reagent. LDH levels were expressed as percentages, according to the formula: cytotoxicity (%) = 100 × Experimental LDH Release absorbance/Maximum LDH Release absorbance (positive control).

### Cell viability: MTT colorimetric assay

Cell viability was evaluated using methylthiazol tetrazolium (MTT) assay according manufacturer instructions. Briefly, 1 × 10^5^ OD-21 cells/well were plated into 96-well cell culture plates and stimulated with LTB_4_-loaded MS or soluble LTB_4_ (Cayman Chemical Company) for 24 h.

The stimuli were removed and 10 µL of MTT (3-(4,5-dymethylthiazol-2-yl)-2,5-diphenyltetrazoluim bromide, Sigma-Aldrich CO., Catalog number M2128) supplemented with 150 µL RPMI (Roswell Park Memorial Institute) medium 1640 (Gibco) was added to the plates. After 3 h incubation, 40 µL of SDS (sodium dodecyl sulphate) buffer was added and cell viability was determined using a SpectraMax® Paradigm® spectrophotometer (Molecular Devices, LLC, Sunnyvale CA, USA). Data obtained was analyzed using a standard curve containing a known number of cells.

### RNA extraction, reverse transcription, and polymerase chain reaction in real time (qRT-PCR)

For evaluation of cell differentiation and biomineralization signaling, integrin binding sialoprotein (*Ibsp)*, runt-related transcription factor 2 (*Runx2*)*,* dentin sialophosphoprotein (*Dspp*) and dentin matrix protein-1 (*Dmp1*) mRNA levels were assayed by quantitative reverse transcription polymerase chain reaction (qRT-PCR). mRNA levels were measured by quantitative reverse transcriptase-polymerase chain reactions (qRT-PCR). To this end, total RNA was extracted using the RNeasy® Mini kit (Qiagen Inc., Valencia, USA) and quantified using NanoDrop 2000 spectrophotometer (Thermo Fisher Scientific Inc., Wilmington, USA). A total of 1 µg of RNA were used for cDNA synthesis with the High Capacity cDNA Reverse Transcription kit (Applied Biosystems, Foster City, USA) in a thermal cycler (Veriti® Thermal Cycler, Applied Biosystems, USA). qRT-PCR reactions were performed in duplicate using the TaqMan® system in a StepOne Plus® real-time PCR system (StepOne Plus® Real-Time PCR System, Applied Biosystems) and the following cycle program: 95 °C for 20 s, 40 cycles at 95 °C for 1 s, and 60 °C for 20 s. Primer–probe pairs were obtained commercially, and thus their sequences are not available (TaqMan**®** Gene Expression Assay, Applied Biosystems). Glyceraldehyde-3-phosphate dehydrogenase (*Gapdh*) was used as reference genes for normalization purposes. The results were analyzed based on cycle threshold (Ct) values. Relative expression was calculated by the ΔΔCt method.

### Biomineralization assay

Mineralized nodule formation was assessed by culturing confluent OD-21 cells in biomineralization media for 28 days with changes of media every third-day. Biomineralization media consisted of DMEM culture media supplemented with 10 mM β-glycerophosphate, 50 μg/ml ascorbic acid, and 1% FBS. OD-21 cells were treated with LTB_4_-MS or mineralizing media alone and with the combination of both. Mineralized monolayer cell cultures were stained for matrix biomineralization as described previously [21]. Briefly, cultures were fixed with 70% ethanol for 10 min and stained with 2% Alizarin Red solution (Sigma) for 5 min at room temperature. To quantify the degree of calcium accumulation in the mineralized extracellular matrix, Alizarin Red-stained cultures were incubated with 100 mM cetylpyridinium chloride (Sigma) for 1 h to release calcium-bound dye into solution. The absorbance of the released dye was measured at 570 nm using a spectrophotometer, and normalized by the total protein concentration in the culture.

### Statistical analysis

Statistical analysis was performed using GraphPad Prism 6 software (GraphPad software Inc., La Jolla, USA). Groups were compared using the one-way ANOVA test followed by Dunnett's post-test (α = 0.05).

## Results

**Fig. 1:**
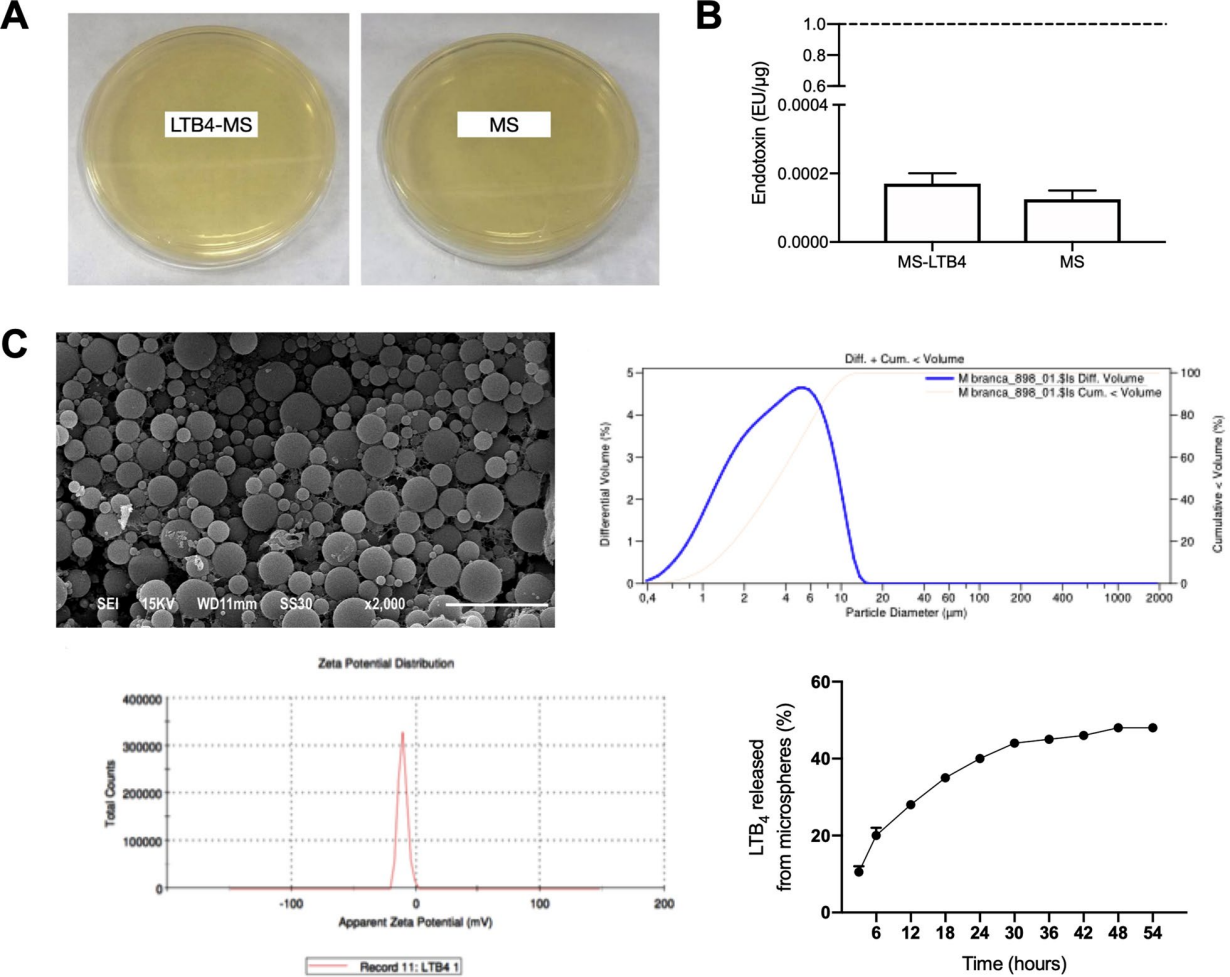
Characterization of PLGA-microspheres. **A** Culture of microspheres containing LTB4 on BHI-agar after 24 h incubation. **B** Data from LPS contamination of microspheres (MS) with or without LTB4. Endotoxins (below 0.1 EU/1 µg of polymer). **C** MEV image, size distribution, zeta potential distribution and in vitro LTB_4_ release assay

PLGA microspheres (loaded with LTB_4_ or empty) exhibited no bacterial growth after 24 h incubation in BHI-agar at 37 °C (Fig. 1A). Also, the endotoxin levels in all samples (encapsulated LTB_4_ or in empty microspheres) were less than 0.1 EU/μg (Fig. 1B).

Microspheres presented similar diameter with average diameter of 5.01 ± 4.4 μm for LTB_4_ loaded MS and 4.53 ± 2.23 μm for unloaded-MS (*p* > 0.05). The zeta potential was  − 12.3 ± 3.49 mV for LTB_4_ loaded MS and − 20.6 ± 4.8 mV for unloaded-MS. In the scanning electron microscopy (SEM) was observed spherical, nonporous and non-aggregated microspheres.

The encapsulation efficiency of LTB_4_ was 39 ± 3.13% (Fig. 1C). Analysis of LTB_4_ release showed a burst release from MS at 6 h, when approximately 20% of the mediator was detected in the medium. After 48 h, 48% of LTB_4_ was released. These results indicate that PLGA biodegradation allows for a progressive release of LTB_4_ up to 54 h (Fig. 1C).

**Fig. 2:**
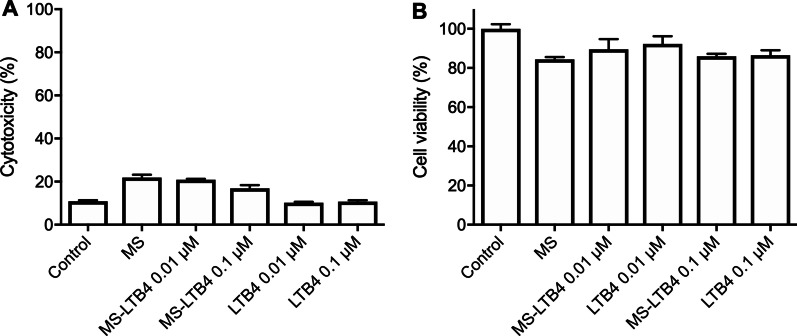
**A** Cytotoxicity using LDH assay in undifferentiated dental pulp cells (OD-21) added to microspheres (MS) with or without LTB_4_ after 24 h. **B** Cell viability of undifferentiated dental pulp cells (OD-21) added to microspheres (MS) with or without LTB_4_ using MTT assay after 24 h

Treatment with empty microspheres or with LTB_4_ 0.01 μM and 0.1 μM showed low cytotoxicity, which was similar to the control (*p* > 0.05) (Fig. 2A). The number of viable cells treated with LTB_4_ encapsulated in microspheres compared to the empty microspheres and LTB_4_ soluble were not statistically significant (*p* > 0.05) (Fig. 2B).

**Fig. 3:**
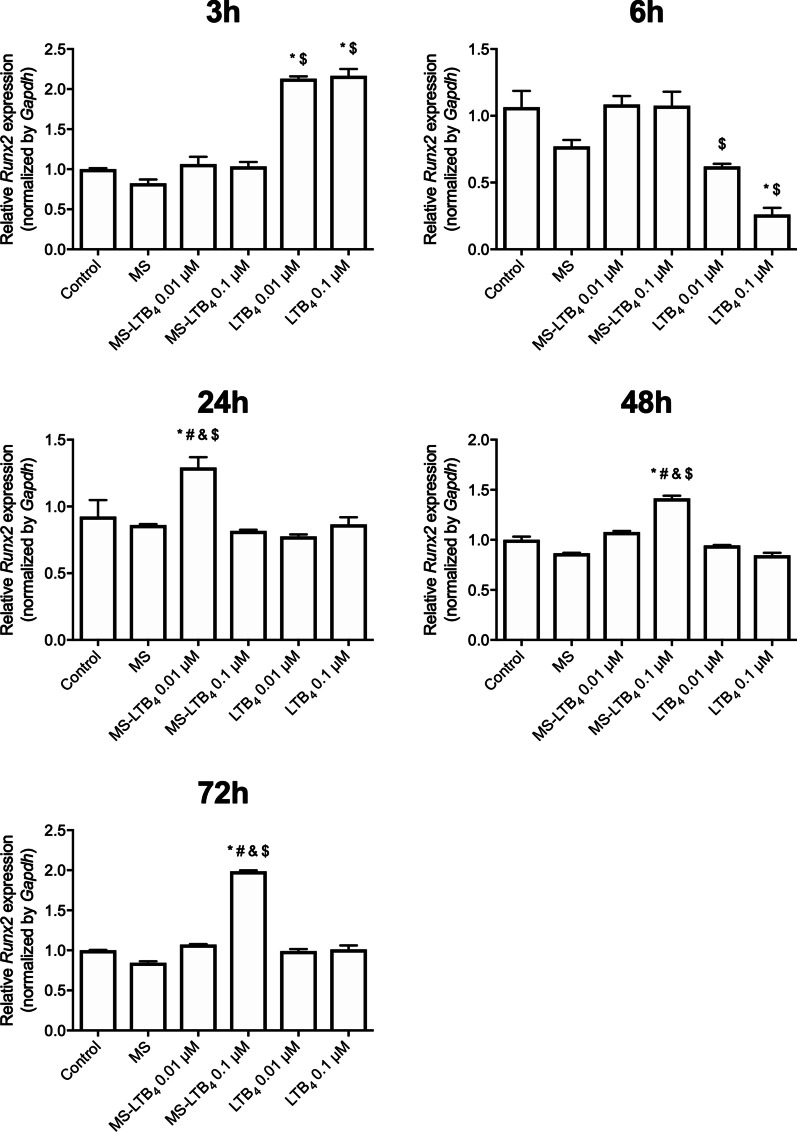
*Runx2* gene expression after stimulation or not with microspheres associated or not with LTB4 on the experimental times of 3, 6, 24, 48 and 72 h. *p < 0.05 compared to control (non-stimulated cells), ^#^*p* < 0.05 compared to empty microspheres, ^&^*p* < 0.05 comparison between MS-LTB4 0.01 µM and 0.1 µM, ^§^*p* < 0.05 comparison between LTB4 0.01 µM and 0.1 µM, and ^$^*p* < 0.05 comparison between soluble and MS at the same concentration

*Runx2* expression increased after a 3 h stimulation period with LTB_4_ in both concentrations (*p* < 0.05). Within 6 h, the non stimulated group and groups of cells stimulated with LTB_4_ microspheres in both molarities had increased *Runx2* expression (*p* < 0.05). At 24 h only the 0.01 µM LTB_4_ microspheres group increased *Runx2* expression (*p* < 0.05). After a stimulation period of 48 and 72 h, the group that received treatment with microspheres with 0.01 µM LTB_4_ showed an increased *Runx2* expression (*p* < 0.05) (Fig. 3).

**Fig. 4:**
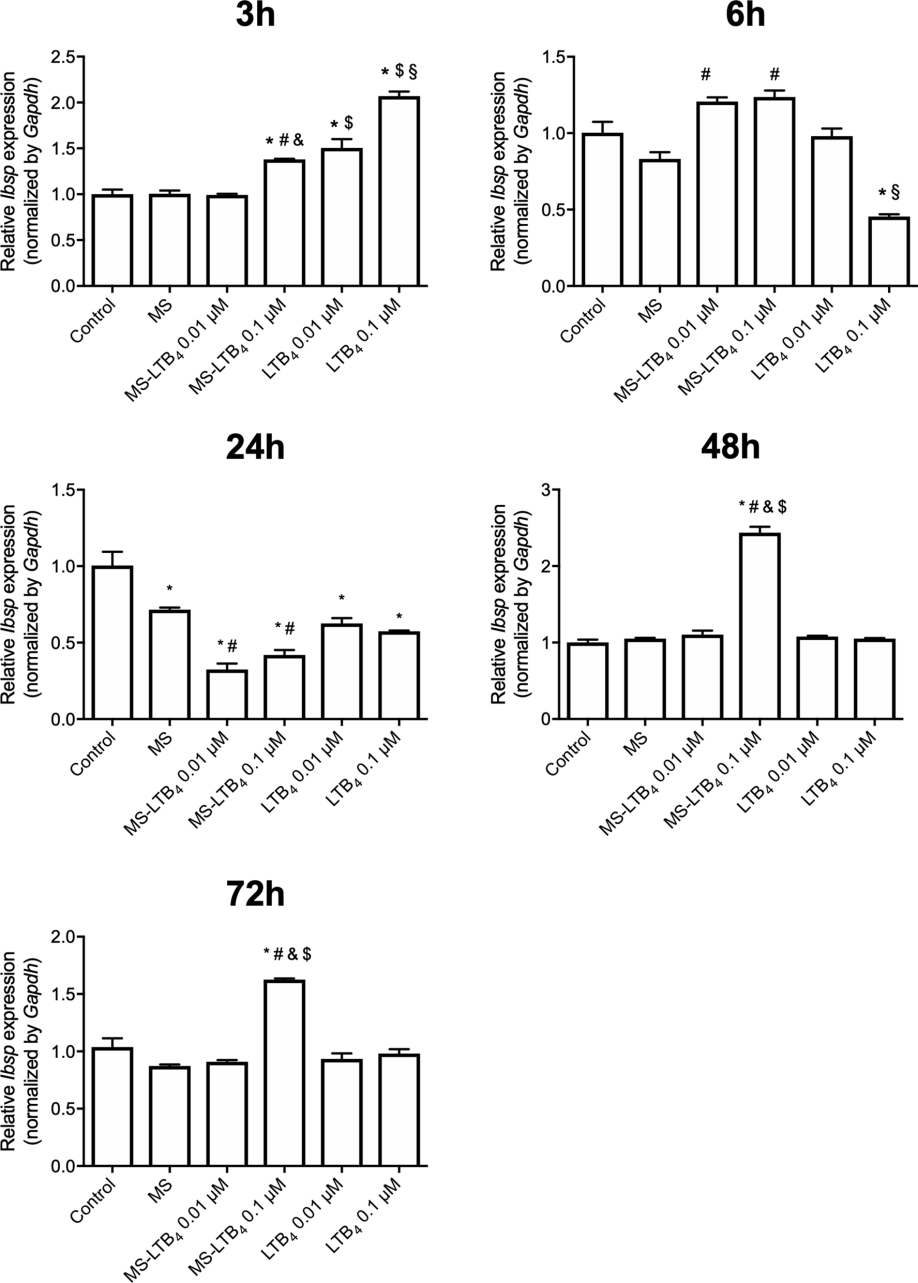
*Ibsp* gene expression after stimulation or not with microspheres associated or not with LTB_4_ on the experimental times of 3, 6, 24, 48 and 72 h. *p < 0.05 compared to control (non-stimulated cells), ^#^*p* < 0.05 compared to empty microspheres, ^&^*p* < 0.05 comparison between MS-LTB_4_ 0.01 µM and 0.1 µM, ^§^*p* < 0.05 comparison between LTB_4_ 0.01 µM and 0.1 µM, and ^$^*p* < 0.05 comparison between soluble and MS at the same concentration

Regarding *Ibsp* gene expression in the early period of time (3 h), the LTB_4_ 0.1 µM showed higher expression of this gene (*p* < 0.05). On the other hand, in the periods of 6, 48 and 72 h, gene expression was higher in group with 0.1 µM LTB_4_ microsphere (*p* < 0.05) (Fig. 4). *Dmp1* and *Dspp* gene expression was not detected in short term culture.

**Fig. 5:**
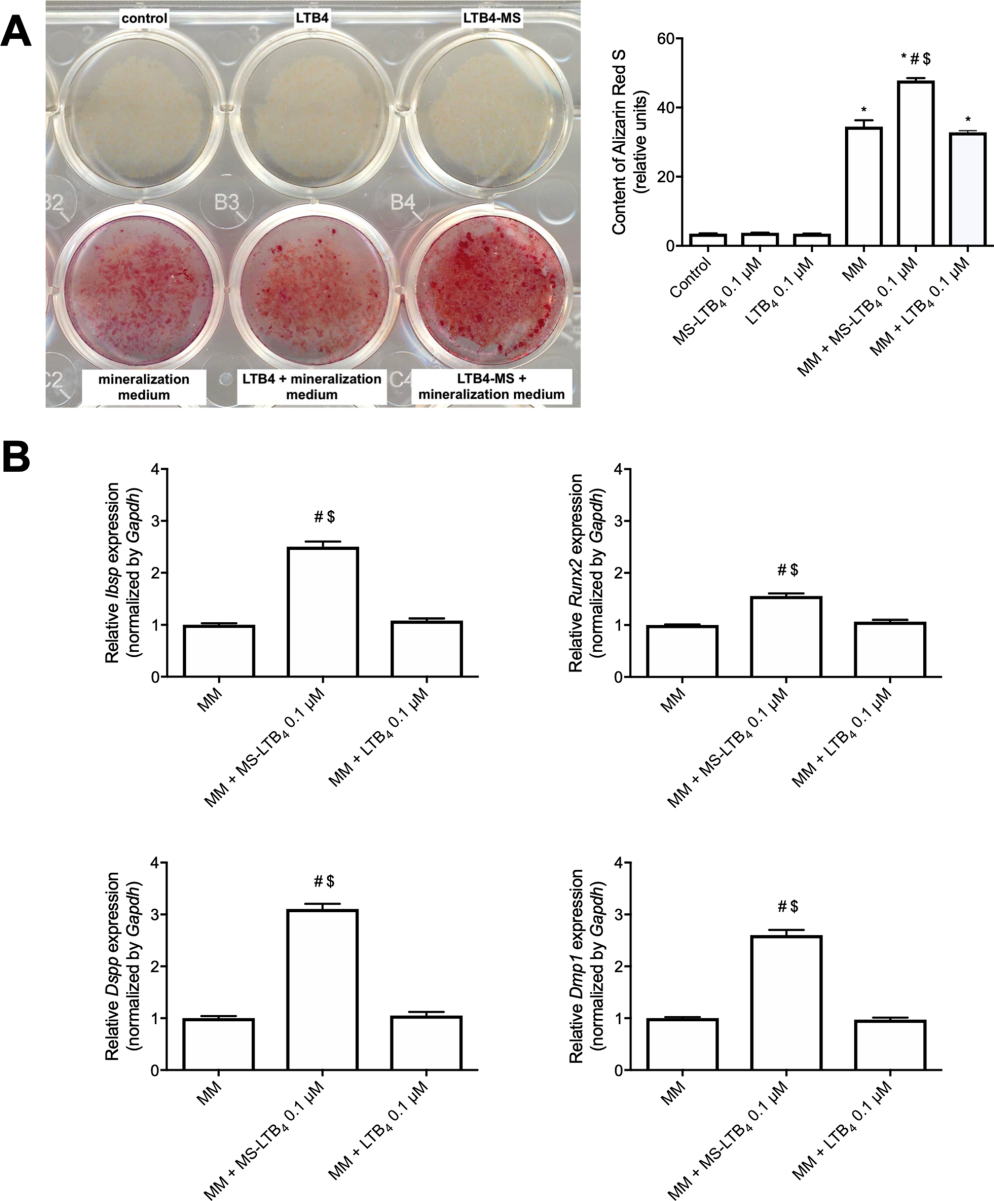
**A** Mineralized nodule formation after stimulation with microspheres associated or not with LTB_4_ for 28 days. **B** *Ibsp*, *Runx2*, *Dspp* and *Dmp1* gene expression after stimulation or not with MS-LTB_4_ for 28 days in biomineralization media. *p < 0.05 compared to control (non-stimulated cells), ^#^*p* < 0.05 compared to biomineralization media alone, ^$^*p* < 0.05 comparison between soluble and MS at the same concentration

To further understand the role of LTB_4_-MS in OD-21 cell differentiation, the ability of cells to produce mineralized nodules was investigated. On day 28, LTB_4_-MS (0.1 µM) induced mineralized nodule formation more than cells maintained in biomineralization media alone (*p* < 0.05). *Ibsp*, *Runx2*, *Dspp* and *Dmp1* gene expression at 28 days were higher in cells treated with LTB_4_-MS (0.1 µM) compared to biomineralization media alone (*p* < 0.05) (Fig. 5).

## Discussion

Here we found that LTB_4_ induced an odontoblastic phenotype in dental pulp cells and production of mineralized nodules. LTB_4_ is a proinflammatory mediator derivate from the enzymatic oxidation of arachidonic acid involved in dental pulp inflammatory reactions [9, 10, 14, 22, 23], but none of them evaluated your effect in the osteogenic and odontogenic differentiation of dental pulp stem cells. Therefore, the null hypothesis was rejected once LTB_4_ loaded in microspheres regulated the expression of genes related to odontoblastic differentiation and biomineralization in mouse dental pulp stem cells.

As LTB_4_ shows a half-life relatively short, in this study the use of microspheres had the aim to preserve its biological activities a longer time and protect the mediator from degradation [24]. LTB_4_ showed no cytotoxic to dental pulp cells, measured by the percentage of cell death of less than 30% and in accordance to the International Organization for Standardization guidelines [25]. Other studies that used the PLGA microspheres demonstrated that it is biocompatible and act as particulate adjuvants [17, 24, 26–29]. All these studies showed that microspheres are a viable way to delivery mediators for prolonged time.

The expression of *Runx2* was upregulated by LTB_4_ soluble after 3 h and after 6, 24, 48 and 72 h by LTB_4_—loaded MS in different concentrations (0.01 and 0,1 μM), indicating the involvement of this mediator in *Runx2* expression [30]. *Runx2* is a transcription factor highly expressed in mesenchymal cells and dental papilla, which is essential for osteoblast and odontoblast differentiation and regulates these cell proliferations [31–33]. Hight doses of LTB_4_ can stimulate the osteoblastic cell proliferation while low doses exhibited an inhibitory effect [34]. In this study, the use of microspheres prolongated the action of LTB_4_ and it may have corroborated to this effect by increasing the expression of *Runx2.*

Integrin binding sialoprotein belongs to a family of proteins, exclusively located in mineralized tissues and crucial for the homeostasis of bone remodeling. The role of this protein involves the initiation of mineral deposition (hydroxyapatite) and increasing of osteoclastogenesis (bone resorption) [35]. In bacterial-induced apical periodontitis, the LTB_4_ is involved in the signaling for osteclastogenesis by the action of leukotriene B4 type 1 receptor (BLT1) [10].

In this study *Ibsp* presented high relative expression after 3 h of stimulation with LTB_4_ soluble, however it decreases in the other times analyzed, 6, 24, 48 and 72 h. While LTB_4_—loaded MS upregulated the expression of *Ibsp* at 48 and 72 h. This upregulation can be associated to high expressions of *Runx2* as some in-vitro studies demonstrated that the expression of bone matrix protein genes, as integrin binding sialoprotein (*Ibsp*) can be upregulated by *Runx2* [33, 36].

Two LTB_4_ receptor have been cloned: BLT1 and BLT2. BLT1 is the high-affinity receptor predominantly expressed in leukocytes and acts as a potent chemotactic receptor for inflammatory cells [15, 37]. LTB_4_ can stimulate the osteoclast differentiation and bone resorption [38] by the activation of LTB_4_/BLT1 mechanism [39]. BLT2 is the low-affinity receptor and has been associated with reduction of pain and wound-healing acceleration by cell proliferation [40]. The prolongated effect of LTB_4_ promoted by the microspheres could activate the LTB_4_/BLT2 mechanism and promote cell proliferation and differentiation. The increase in the relative expression of *Runx2* and *Ibsp* might be related to that as BLT2 plays an important role in the wound- healing by cell proliferation [18].

A recent study demonstrated that LTB_4_ needs an incubation time of 24 h to assure an adequate ligation with the receptor and present the intended pharmacological effects, as accelerated wound-healing rate [40]. Therefore, the use of microspheres can be a strategy to preserve the biological activities of the mediator for prolonged times and activated this receptor. One should not expect a direct correlation between in vitro and in vivo concentration of mediators released from microspheres, specially because the environment might influence that, due to inflammation, edema, dilution, etc. In this preclinical in vitro study, cell differentiation under LTB_4_ stimuli was investigated. Later on, in vivo investigation should be performed to optimize the deliver to in vivo preclinical and clinical studies.

There are several clinical procedures that the materials can be directly applied to dental pulp which includes direct pulp capping, partial pulpotomy or full pulpotomy. Our findings shed light on a novel pharmacological strategy to delivery stimuli capable of inducing differentiation of dental pulp cells. Because LTB_4_-MS can efficiently drive OD-21 cells into an odontoblast phenotype, these findings opens the avenue for a future clinical application. One limitation of our study is that the results were obtained in an in vitro study, requiring further in vivo investigation.

## Conclusion

LTB_4_, soluble or loaded in MS, were not cytotoxic and modulated the expression of the *Ibsp* and *Runx2* genes in cultured OD-21 cells. When LTB_4_ was incorporated into MS, odontoblast differentiation and mineralization was induced in long term culture. Our findings shed light on a novel pharmacological strategy to delivery stimuli capable of inducing differentiation of dental pulp cells obtained from a mouse cell lineage.

## Data Availability

The datasets used and/or analysed during the current study are available from the corresponding author on reasonable request.

## Abbreviations

LTB_4_: Leukotriene B_4_
MS: Microspheres
LDH: Lactate dehydrogenase
MTT Assay: Methylthiazol tetrazolium (MTT) assay
μM: Micrometer
OD-21: Dental pulp cells
*Ibsp*: Integrin binding sialoprotein
*Runx2*: Runt-related transcription factor 2
LT: Leukotrienes
FLAP: 5-Lipoxygenase activating protein
5-LO: 5-Lipoxygenase
5S-HpETE: 5S-hydroxyperoeicosatetraenoic acid
5S-HETE: 5S acid-hydroxyieicositetraenoic
LTA4: Leukotriene A4
BLT1: Leukotriene receptor 1
BLT2: Leukotriene receptor 2
PLGA: Lactic-co-glycolic acid
°C: Degrees celsius
µL: Microliter
PBS: Phosphate Buffered Saline
BHI: Brain Heart Infusion
LAL: Limulus Amebocyte Lysate
EU/mL: Endotoxin units per milliliter
mg: Milligram
mL: Milliliter
SEM: Scanning electron microscopy
h: Hour
DMEM: Dulbecco's Modified Eagle's Medium
FBS: Fetal bovine serum
PBS: Phosphate buffered saline
RPMI: Roswell Park Memorial Institute
SDS: Sodium dodecyl sulphate
*Dspp*: Dentin sialophosphoprotein
*Dmp1*: dentin matrix protein-1
µg: Microgram
*Gapdh*: Glyceraldehyde-3-phosphate dehydrogenase
Ct: Cycle threshold
nm: Nanometer
